# Di-μ-aqua-bis­{diaqua­[μ-4-({4-[bis­(2-hy­droxy­eth­yl)amino]-6-chloro-1,3,5-triazin-2-yl}amino)­benzene­sulfonato]­sodium(I)}

**DOI:** 10.1107/S1600536812023732

**Published:** 2012-05-31

**Authors:** Mei Zhang, Fu-Yu Sun, Gui-Zhe Zhao, Ya-Qing Liu

**Affiliations:** a Research Center for Engineering Technology of Polymeric Composites of Shanxi Province, College of Materials Science and Engineering, North University of China, Taiyuan 030051, People’s Republic of China

## Abstract

In the dinuclear title compound, [Na_2_(C_13_H_15_ClN_5_O_5_S)_2_(H_2_O)_6_]_*n*_, two Na^+^ cations, disposed about a centre of inversion, are linked by two bridging water mol­ecules. The coordination geometry is based on an O_5_ donor set defined by four water mol­ecules and a 4-amino­benzene­sulfonate O atom in a distorted trigonal–bipyramidal geometry. In the crystal, significant O—H⋯O, O—H⋯N and N—H⋯O hydrogen bonds lead to the formation of a three-dimensional architecture.

## Related literature

For commercial and synthetic applications of related compounds, see: Candiani & Frigerio (2007 ▶); Hollink *et al.* (2005 ▶); Konstantion & Petrova (2002 ▶).
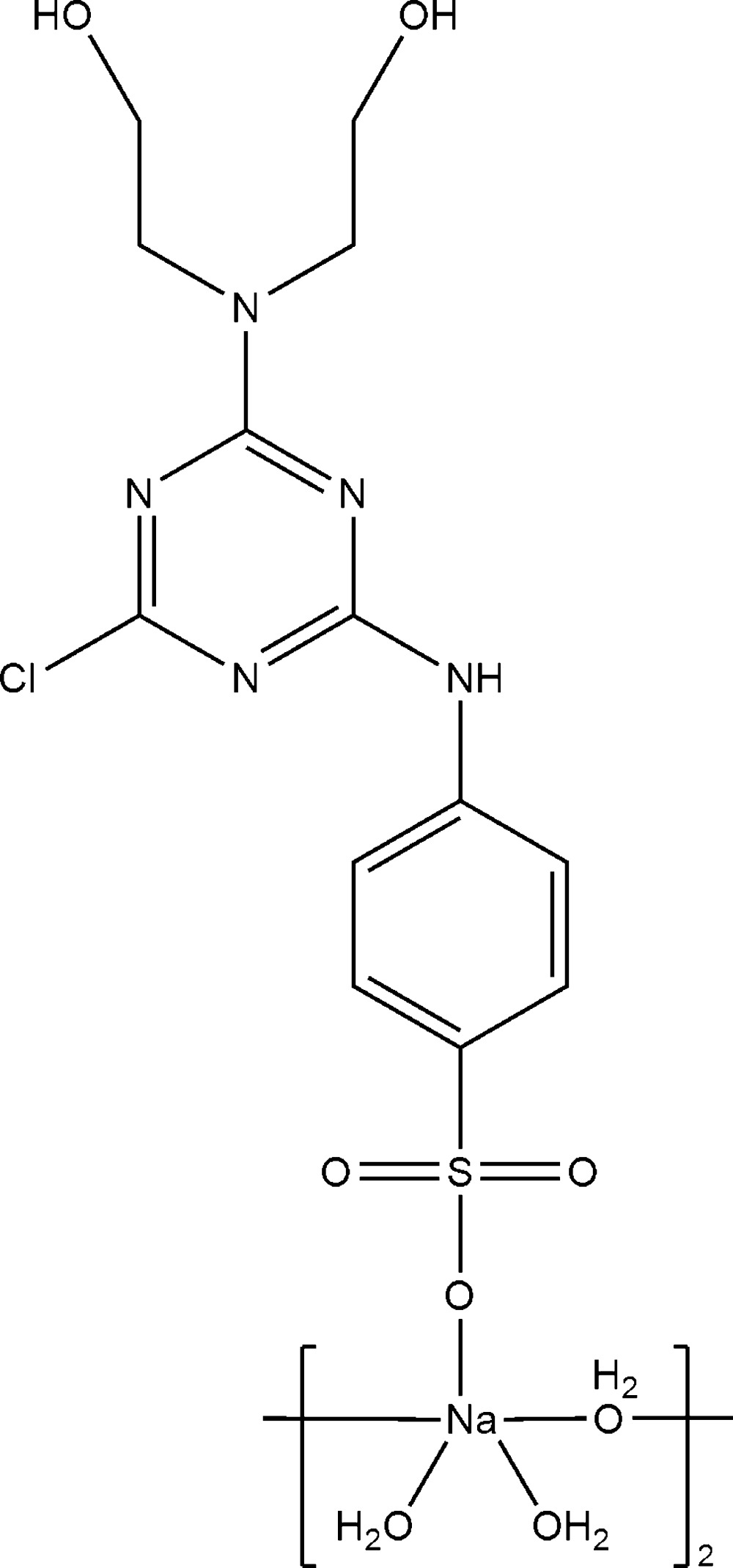


## Experimental

### 

#### Crystal data

[Na_2_(C_13_H_15_ClN_5_O_5_S)_2_(H_2_O)_6_]
*M* *_r_* = 931.72
Triclinic, 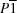

*a* = 7.5628 (7) Å
*b* = 8.6274 (8) Å
*c* = 15.532 (2) Å
α = 97.348 (2)°
β = 93.363 (4)°
γ = 102.410 (7)°
*V* = 977.75 (18) Å^3^
*Z* = 1
Mo *K*α radiation
μ = 0.38 mm^−1^
*T* = 113 K
0.50 × 0.04 × 0.04 mm

#### Data collection

Rigaku Saturn724 CCD diffractometer
Absorption correction: multi-scan (*CrystalClear*; Molecular Structure Corporation & Rigaku, 2005 ▶) *T*_min_ = 0.834, *T*_max_ = 0.985
8262 measured reflections
3418 independent reflections
1459 reflections with *I* > 2σ(*I*)
*R*_int_ = 0.106

#### Refinement

*R*[*F* ^2^ > 2σ(*F* ^2^)] = 0.062
*wR*(*F* ^2^) = 0.131
*S* = 0.87
3418 reflections
283 parameters
20 restraints
H atoms treated by a mixture of independent and constrained refinement
Δρ_max_ = 0.52 e Å^−3^
Δρ_min_ = −0.52 e Å^−3^

### 

Data collection: *CrystalClear* (Molecular Structure Corporation & Rigaku, 2005 ▶); cell refinement: *CrystalClear*; data reduction: *CrystalClear*; program(s) used to solve structure: *SHELXS97* (Sheldrick, 2008 ▶); program(s) used to refine structure: *SHELXL97* (Sheldrick, 2008 ▶); molecular graphics: *SHELXTL* (Sheldrick, 2008 ▶); software used to prepare material for publication: *CrystalClear*.

## Supplementary Material

Crystal structure: contains datablock(s) global, I. DOI: 10.1107/S1600536812023732/tk5096sup1.cif

Structure factors: contains datablock(s) I. DOI: 10.1107/S1600536812023732/tk5096Isup2.hkl

Additional supplementary materials:  crystallographic information; 3D view; checkCIF report

## Figures and Tables

**Table 1:** Hydrogen-bond geometry (Å, °)

*D*—H⋯*A*	*D*—H	H⋯*A*	*D*⋯*A*	*D*—H⋯*A*
O1—H1⋯N4^i^	0.87 (2)	1.96 (3)	2.823 (5)	172 (5)
O2—H2⋯O3^ii^	0.86 (3)	1.94 (3)	2.772 (4)	165 (4)
O6—H6*A*⋯O5^iii^	0.99	1.83	2.817 (4)	174
O6—H6*B*⋯N3^iv^	0.99	2.23	3.009 (5)	135
O7—H7*A*⋯O8^v^	0.87 (2)	2.02 (3)	2.861 (5)	164 (4)
O7—H7*B*⋯O2^ii^	0.82 (2)	1.95 (2)	2.767 (4)	169 (5)
O8—H8*A*⋯O5^vi^	0.80 (2)	2.03 (3)	2.797 (4)	162 (4)
O8—H8*B*⋯O3^iii^	0.82 (2)	2.15 (3)	2.907 (5)	154 (4)
N5—H5⋯O1^iv^	0.90 (4)	2.01 (4)	2.828 (5)	151 (4)

Symmetry codes: (i) 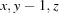; (ii) 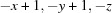; (iii) 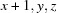; (iv) 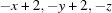; (v) 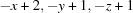; (vi) 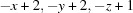.

**Fig. 1.**
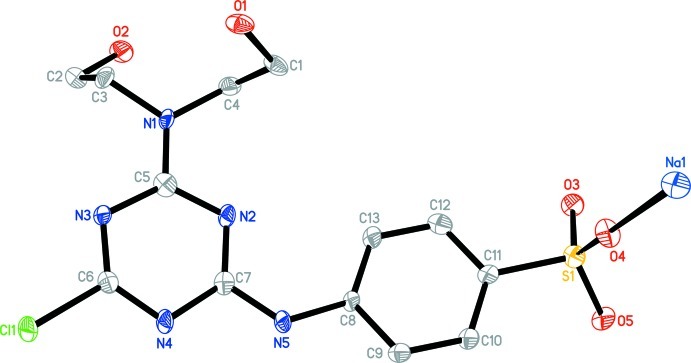
The molecular structure of (I). Displacement ellipsoids are drawn at the 30% probability level. H atoms have been omitted.

**Fig. 2.**
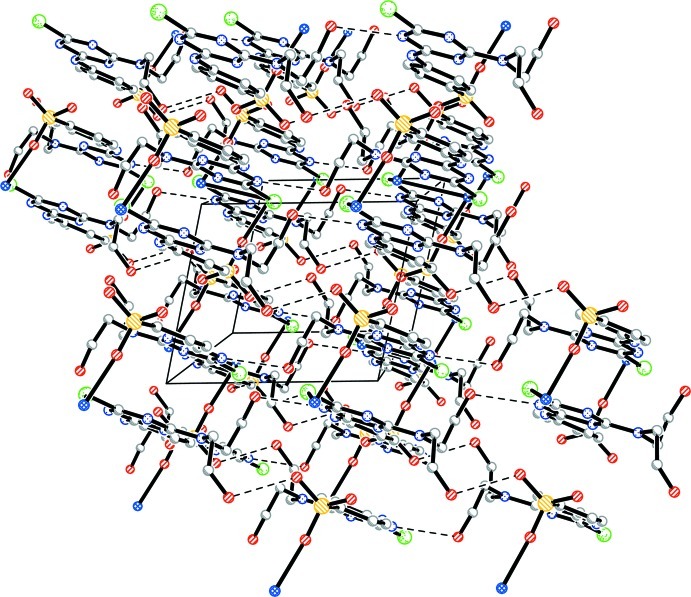
A view of the crystal packing for (I). Hydrogen bonds are shown as dashed lines.

## supplementary crystallographic information

### Comment

Cyanuric chloride derivatives are widely used in commercial chemicals, especially in pesticides, reactive dyes, fluorescent brighteners, liposome and polymer photostabilizers (Hollink *et al.*, 2005; Candiani & Frigerio, 2007). The widespread use of these compounds is due to their higher reactive activity (Konstantion & Petrova, 2002). The title compound belongs to the cyanuric chloride derivatives and its structure is reported herein.

The asymmetric unit is shown in Fig. 1. The dihedral angle between the benzene ring and the triazine ring is 8.6 (2)°. As shown in Fig. 2, the crystal packing displays O—H···O, O—H···N and N—H···O hydrogen bonds, Table 1.

### Experimental

Cyanuric chloride (0.1 mol) was dissolved in acetone (120 ml). This solution was poured into distilled water (150 ml) with crushed ice (150 g). The reaction system was stirred maintaining the temperature at 0–5 °C in the ice-bath. An aqueous solution of sodium sulfate (0.1 mol) was slowly dropped into the above reaction vessel within 0.5 h, and then a 20% aqueous solution of sodium carbonate was added drop-wise to the reaction mixture to keep the pH at 7–8. Then the mixture was kept stirring for 5 h at 0–5 °C. After the reaction was completed, the white precipitate was filtered, washed with acetone and water twice, respectively, and dried at room temperatuer under vacuum to constant weight. This white powder is intermediate I. The intermediate I (0.05 mol) and a mixed solution of water and acetone (160 ml) were added into 250 ml four-neck flask. After stirring for 0.5 h at 25 °C, an aqueous solution of diethanol amine (0.06 mol) was slowly dropped into the reaction vessel within 0.5 h, and then a 20% aqueous solution of sodium carbonate was added drop-wise to the reaction mixture to keep the pH at 8–9. Then the mixture was kept stirring for 6 h at 45 °C. After the reaction was completed, the solution was rotary evaporated, washed with anhydrous alcohol twice, and dried at room temperature under vacuum to constant weight. The target product was obtained. Crystals of the title compound were obtained by slow evaporation of its methanol/*n*-hexane solution held at room temperature.

### Refinement

Carbon-bound H-atoms were placed in calculated positions [C—H = 0.95 to 0.99 Å, *U*_iso_(H) = 1.2*U*_eq_(C)] and were included in the refinement in the riding model approximation. The positions of the O- and N- bound H-atoms were refined with light distance restraints, and with *U*_iso_(H) = 1.2*U*_eq_(water-O,N) and *U*_iso_(H) = 1.5*U*_eq_(hydroxyl-O)].

### Crystal data

**Table:**

[Na_2_(C_13_H_15_ClN_5_O_5_S)_2_(H_2_O)_6_]	*Z* = 1
*M**_r_* = 931.72	*F*(000) = 484
Triclinic, *P*1	*D*_x_ = 1.582 Mg m^−^^3^
Hall symbol: -P 1	Mo *K*α radiation, λ = 0.71073 Å
*a* = 7.5628 (7) Å	Cell parameters from 3065 reflections
*b* = 8.6274 (8) Å	θ = 2.4–26.1°
*c* = 15.532 (2) Å	µ = 0.38 mm^−^^1^
α = 97.348 (2)°	*T* = 113 K
β = 93.363 (4)°	Prism, colourless
γ = 102.410 (7)°	0.50 × 0.04 × 0.04 mm
*V* = 977.75 (18) Å^3^	

### Data collection

**Table:**

Rigaku Saturn724 CCD diffractometer	3418 independent reflections
Radiation source: rotating anode	1459 reflections with *I* > 2σ(*I*)
Multilayer monochromator	*R*_int_ = 0.106
Detector resolution: 14.22 pixels mm^-1^	θ_max_ = 25.0°, θ_min_ = 2.4°
ω and φ scans	*h* = −8→8
Absorption correction: multi-scan (*CrystalClear*; Molecular Structure Corporation & Rigaku, 2005)	*k* = −10→10
*T*_min_ = 0.834, *T*_max_ = 0.985	*l* = −18→18
8262 measured reflections	

### Refinement

**Table:**

Refinement on *F*^2^	Primary atom site location: structure-invariant direct methods
Least-squares matrix: full	Secondary atom site location: difference Fourier map
*R*[*F*^2^ > 2σ(*F*^2^)] = 0.062	Hydrogen site location: inferred from neighbouring sites
*wR*(*F*^2^) = 0.131	H atoms treated by a mixture of independent and constrained refinement
*S* = 0.87	*w* = 1/[σ^2^(*F*_o_^2^) + (0.0293*P*)^2^] where *P* = (*F*_o_^2^ + 2*F*_c_^2^)/3
3418 reflections	(Δ/σ)_max_ = 0.001
283 parameters	Δρ_max_ = 0.52 e Å^−^^3^
20 restraints	Δρ_min_ = −0.52 e Å^−^^3^

### Special details

**Table:**

Geometry. All e.s.d.'s (except the e.s.d. in the dihedral angle between two l.s. planes) are estimated using the full covariance matrix. The cell e.s.d.'s are taken into account individually in the estimation of e.s.d.'s in distances, angles and torsion angles; correlations between e.s.d.'s in cell parameters are only used when they are defined by crystal symmetry. An approximate (isotropic) treatment of cell e.s.d.'s is used for estimating e.s.d.'s involving l.s. planes.
Refinement. Refinement of *F*^2^ against ALL reflections. The weighted *R*-factor *wR* and goodness of fit *S* are based on *F*^2^, conventional *R*-factors *R* are based on *F*, with *F* set to zero for negative *F*^2^. The threshold expression of *F*^2^ > σ(*F*^2^) is used only for calculating *R*-factors(gt) *etc*. and is not relevant to the choice of reflections for refinement. *R*-factors based on *F*^2^ are statistically about twice as large as those based on *F*, and *R*- factors based on ALL data will be even larger.

### Fractional atomic coordinates and isotropic or equivalent isotropic displacement parameters (Å^2^)

**Table:**

	*x*	*y*	*z*	*U*_iso_*/*U*_eq_	
Na1	0.9529 (2)	0.8060 (2)	0.46061 (12)	0.0216 (5)	
S1	0.55329 (16)	0.91677 (15)	0.34804 (8)	0.0191 (3)	
Cl1	1.02875 (15)	1.27203 (14)	−0.25219 (8)	0.0221 (4)	
O1	0.9492 (4)	0.5459 (4)	−0.0875 (2)	0.0213 (9)	
H1	0.934 (6)	0.443 (3)	−0.099 (3)	0.032*	
O2	0.4874 (4)	0.5163 (4)	−0.3219 (2)	0.0234 (9)	
H2	0.504 (6)	0.421 (3)	−0.330 (3)	0.035*	
O3	0.4056 (4)	0.7718 (3)	0.32366 (19)	0.0210 (8)	
O4	0.7162 (4)	0.8814 (4)	0.3889 (2)	0.0216 (9)	
O5	0.4936 (4)	1.0469 (4)	0.40039 (19)	0.0194 (8)	
N1	0.7584 (5)	0.7287 (4)	−0.1875 (2)	0.0161 (10)	
N2	0.7670 (5)	0.9391 (4)	−0.0807 (2)	0.0162 (10)	
N3	0.8817 (5)	0.9841 (4)	−0.2196 (2)	0.0150 (9)	
N4	0.8923 (5)	1.2084 (4)	−0.1092 (2)	0.0175 (10)	
N5	0.7900 (5)	1.1689 (5)	0.0233 (3)	0.0170 (10)	
H5	0.851 (5)	1.272 (5)	0.029 (3)	0.020*	
C1	0.7906 (5)	0.5903 (5)	−0.0571 (3)	0.0195 (12)	
H1A	0.8270	0.6892	−0.0142	0.023*	
H1B	0.7234	0.5042	−0.0271	0.023*	
C2	0.6672 (6)	0.6188 (5)	−0.1302 (3)	0.0207 (12)	
H2A	0.6108	0.5145	−0.1660	0.025*	
H2B	0.5682	0.6632	−0.1048	0.025*	
C3	0.8018 (6)	0.6556 (5)	−0.2719 (3)	0.0192 (12)	
H3A	0.8331	0.5519	−0.2650	0.023*	
H3B	0.9100	0.7264	−0.2901	0.023*	
C4	0.6466 (6)	0.6270 (5)	−0.3431 (3)	0.0227 (13)	
H4A	0.6151	0.7303	−0.3507	0.027*	
H4B	0.6862	0.5828	−0.3989	0.027*	
C5	0.8033 (6)	0.8880 (6)	−0.1625 (3)	0.0189 (12)	
C6	0.9213 (6)	1.1378 (5)	−0.1863 (3)	0.0169 (12)	
C7	0.8149 (6)	1.0990 (6)	−0.0576 (3)	0.0158 (12)	
C8	0.7265 (6)	1.0979 (5)	0.0957 (3)	0.0130 (11)	
C9	0.7404 (6)	1.2041 (5)	0.1720 (3)	0.0180 (12)	
H9	0.7876	1.3154	0.1720	0.022*	
C10	0.6857 (6)	1.1488 (5)	0.2486 (3)	0.0180 (12)	
H10	0.6997	1.2221	0.3010	0.022*	
C11	0.6109 (6)	0.9878 (5)	0.2493 (3)	0.0149 (11)	
C12	0.5925 (6)	0.8800 (6)	0.1722 (3)	0.0203 (12)	
H12	0.5421	0.7691	0.1720	0.024*	
C13	0.6486 (5)	0.9361 (5)	0.0955 (3)	0.0166 (11)	
H13	0.6335	0.8634	0.0428	0.020*	
O6	1.1240 (4)	1.0471 (3)	0.41494 (19)	0.0191 (8)	
H6A	1.2558	1.0514	0.4142	0.023*	
H6B	1.0732	1.0718	0.3594	0.023*	
O7	0.8355 (4)	0.5359 (4)	0.4260 (2)	0.0349 (11)	
H7A	0.825 (6)	0.466 (5)	0.462 (2)	0.042*	
H7B	0.747 (5)	0.518 (5)	0.390 (2)	0.042*	
O8	1.2365 (4)	0.7394 (4)	0.4857 (2)	0.0226 (9)	
H8A	1.297 (5)	0.813 (4)	0.518 (2)	0.027*	
H8B	1.267 (5)	0.717 (5)	0.4365 (17)	0.027*	

### Atomic displacement parameters (Å^2^)

**Table:**

	*U*^11^	*U*^22^	*U*^33^	*U*^12^	*U*^13^	*U*^23^
Na1	0.0141 (10)	0.0229 (12)	0.0293 (12)	0.0041 (9)	0.0094 (9)	0.0061 (9)
S1	0.0148 (7)	0.0226 (8)	0.0217 (8)	0.0051 (6)	0.0093 (6)	0.0052 (6)
Cl1	0.0211 (7)	0.0231 (8)	0.0241 (8)	0.0047 (6)	0.0125 (6)	0.0067 (6)
O1	0.0155 (18)	0.0163 (19)	0.035 (2)	0.0071 (17)	0.0074 (16)	0.0067 (18)
O2	0.0204 (19)	0.019 (2)	0.028 (2)	−0.0016 (18)	0.0032 (16)	0.0022 (18)
O3	0.0175 (18)	0.020 (2)	0.024 (2)	−0.0023 (16)	0.0067 (16)	0.0032 (16)
O4	0.0116 (17)	0.033 (2)	0.026 (2)	0.0147 (16)	0.0025 (15)	0.0102 (16)
O5	0.0145 (18)	0.024 (2)	0.022 (2)	0.0094 (15)	0.0118 (15)	0.0004 (15)
N1	0.018 (2)	0.016 (2)	0.014 (2)	0.0025 (19)	0.0096 (18)	0.0017 (18)
N2	0.013 (2)	0.021 (2)	0.015 (2)	0.0022 (19)	0.0068 (18)	0.0026 (19)
N3	0.012 (2)	0.015 (2)	0.018 (2)	0.0017 (18)	0.0083 (18)	0.0018 (18)
N4	0.015 (2)	0.022 (3)	0.016 (2)	0.002 (2)	0.0080 (19)	0.0045 (19)
N5	0.016 (2)	0.018 (2)	0.016 (2)	−0.0009 (19)	0.0084 (19)	0.004 (2)
C1	0.015 (3)	0.016 (3)	0.030 (3)	0.004 (2)	0.008 (2)	0.010 (2)
C2	0.023 (3)	0.016 (3)	0.026 (3)	0.008 (2)	0.009 (2)	0.004 (2)
C3	0.019 (3)	0.019 (3)	0.019 (3)	0.004 (2)	0.009 (2)	0.000 (2)
C4	0.028 (3)	0.018 (3)	0.023 (3)	0.005 (3)	0.005 (2)	0.006 (2)
C5	0.009 (3)	0.023 (3)	0.025 (3)	0.002 (2)	0.002 (2)	0.005 (2)
C6	0.015 (3)	0.020 (3)	0.020 (3)	0.009 (2)	0.004 (2)	0.007 (2)
C7	0.008 (3)	0.023 (3)	0.018 (3)	0.007 (2)	0.002 (2)	0.004 (2)
C8	0.009 (3)	0.018 (3)	0.014 (3)	0.007 (2)	0.007 (2)	0.003 (2)
C9	0.016 (3)	0.015 (3)	0.024 (3)	0.003 (2)	0.010 (2)	0.001 (2)
C10	0.017 (3)	0.018 (3)	0.020 (3)	0.004 (2)	0.013 (2)	0.002 (2)
C11	0.011 (2)	0.017 (3)	0.020 (3)	0.009 (2)	0.010 (2)	0.002 (2)
C12	0.016 (3)	0.018 (3)	0.028 (3)	0.004 (2)	0.005 (2)	0.005 (2)
C13	0.013 (3)	0.021 (3)	0.017 (3)	0.004 (2)	0.007 (2)	0.003 (2)
O6	0.0091 (17)	0.030 (2)	0.021 (2)	0.0056 (15)	0.0023 (15)	0.0114 (16)
O7	0.021 (2)	0.034 (3)	0.048 (3)	−0.0018 (19)	−0.0035 (19)	0.017 (2)
O8	0.018 (2)	0.026 (2)	0.022 (2)	0.0009 (17)	0.0065 (17)	0.0023 (18)

### Geometric parameters (Å, º)

**Table:**

Na1—O7	2.290 (4)	N5—H5	0.90 (4)
Na1—O4	2.306 (3)	C1—C2	1.505 (5)
Na1—O6^i^	2.346 (4)	C1—H1A	0.9900
Na1—O8	2.361 (4)	C1—H1B	0.9900
Na1—O6	2.415 (3)	C2—H2A	0.9900
Na1—Cl1^ii^	3.239 (2)	C2—H2B	0.9900
Na1—Na1^i^	3.322 (4)	C3—C4	1.522 (5)
Na1—H8B	2.68 (4)	C3—H3A	0.9900
S1—O4	1.461 (3)	C3—H3B	0.9900
S1—O5	1.467 (3)	C4—H4A	0.9900
S1—O3	1.478 (3)	C4—H4B	0.9900
S1—C11	1.766 (5)	C8—C9	1.386 (6)
Cl1—C6	1.746 (5)	C8—C13	1.393 (6)
Cl1—Na1^ii^	3.238 (2)	C9—C10	1.391 (6)
O1—C1	1.423 (5)	C9—H9	0.9500
O1—H1	0.87 (2)	C10—C11	1.384 (6)
O2—C4	1.451 (5)	C10—H10	0.9500
O2—H2	0.86 (3)	C11—C12	1.400 (6)
N1—C5	1.343 (5)	C12—C13	1.397 (6)
N1—C3	1.467 (5)	C12—H12	0.9500
N1—C2	1.474 (5)	C13—H13	0.9500
N2—C7	1.344 (5)	O6—Na1^i^	2.346 (4)
N2—C5	1.354 (6)	O6—H6A	0.9900
N3—C6	1.323 (5)	O6—H6B	0.9900
N3—C5	1.365 (6)	O7—H7A	0.87 (2)
N4—C6	1.324 (5)	O7—H7B	0.82 (2)
N4—C7	1.374 (5)	O8—H8A	0.80 (2)
N5—C7	1.364 (6)	O8—H8B	0.82 (2)
N5—C8	1.408 (5)		
			
O7—Na1—O4	95.45 (13)	C1—C2—H2A	108.7
O7—Na1—O6^i^	121.20 (14)	N1—C2—H2B	108.7
O4—Na1—O6^i^	85.08 (12)	C1—C2—H2B	108.7
O7—Na1—O8	86.24 (13)	H2A—C2—H2B	107.6
O4—Na1—O8	159.53 (15)	N1—C3—C4	113.2 (4)
O6^i^—Na1—O8	111.51 (12)	N1—C3—H3A	108.9
O7—Na1—O6	146.92 (15)	C4—C3—H3A	108.9
O4—Na1—O6	81.63 (11)	N1—C3—H3B	108.9
O6^i^—Na1—O6	91.52 (12)	C4—C3—H3B	108.9
O8—Na1—O6	85.87 (12)	H3A—C3—H3B	107.7
O7—Na1—Cl1^ii^	74.72 (11)	O2—C4—C3	111.2 (4)
O4—Na1—Cl1^ii^	70.57 (9)	O2—C4—H4A	109.4
O6^i^—Na1—Cl1^ii^	152.67 (10)	C3—C4—H4A	109.4
O8—Na1—Cl1^ii^	90.36 (10)	O2—C4—H4B	109.4
O6—Na1—Cl1^ii^	73.26 (9)	C3—C4—H4B	109.4
O7—Na1—Na1^i^	167.15 (14)	H4A—C4—H4B	108.0
O4—Na1—Na1^i^	80.42 (10)	N1—C5—N2	115.8 (5)
O6^i^—Na1—Na1^i^	46.62 (8)	N1—C5—N3	118.7 (5)
O8—Na1—Na1^i^	101.92 (11)	N2—C5—N3	125.5 (4)
O6—Na1—Na1^i^	44.91 (8)	N3—C6—N4	130.0 (5)
Cl1^ii^—Na1—Na1^i^	114.70 (8)	N3—C6—Cl1	116.6 (4)
O7—Na1—H8B	82.4 (8)	N4—C6—Cl1	113.4 (3)
O4—Na1—H8B	142.8 (7)	N2—C7—N5	121.3 (5)
O6^i^—Na1—H8B	127.8 (6)	N2—C7—N4	125.9 (5)
O8—Na1—H8B	17.3 (6)	N5—C7—N4	112.8 (4)
O6—Na1—H8B	80.6 (9)	C9—C8—C13	119.3 (4)
Cl1^ii^—Na1—H8B	73.1 (6)	C9—C8—N5	114.7 (4)
Na1^i^—Na1—H8B	108.3 (8)	C13—C8—N5	126.0 (4)
O4—S1—O5	112.25 (18)	C8—C9—C10	120.4 (4)
O4—S1—O3	112.36 (19)	C8—C9—H9	119.8
O5—S1—O3	112.56 (18)	C10—C9—H9	119.8
O4—S1—C11	107.4 (2)	C11—C10—C9	120.6 (4)
O5—S1—C11	105.8 (2)	C11—C10—H10	119.7
O3—S1—C11	105.9 (2)	C9—C10—H10	119.7
C6—Cl1—Na1^ii^	125.53 (17)	C10—C11—C12	119.4 (4)
C1—O1—H1	112 (3)	C10—C11—S1	120.3 (4)
C4—O2—H2	110 (3)	C12—C11—S1	120.2 (4)
S1—O4—Na1	173.9 (2)	C13—C12—C11	119.7 (4)
C5—N1—C3	121.7 (4)	C13—C12—H12	120.1
C5—N1—C2	121.4 (4)	C11—C12—H12	120.1
C3—N1—C2	116.9 (4)	C8—C13—C12	120.5 (4)
C7—N2—C5	114.2 (4)	C8—C13—H13	119.8
C6—N3—C5	112.4 (4)	C12—C13—H13	119.8
C6—N4—C7	111.9 (4)	Na1^i^—O6—Na1	88.48 (12)
C7—N5—C8	129.8 (4)	Na1^i^—O6—H6A	113.9
C7—N5—H5	106 (3)	Na1—O6—H6A	113.9
C8—N5—H5	122 (3)	Na1^i^—O6—H6B	113.9
O1—C1—C2	112.1 (4)	Na1—O6—H6B	113.9
O1—C1—H1A	109.2	H6A—O6—H6B	111.1
C2—C1—H1A	109.2	Na1—O7—H7A	126 (3)
O1—C1—H1B	109.2	Na1—O7—H7B	111 (3)
C2—C1—H1B	109.2	H7A—O7—H7B	112 (3)
H1A—C1—H1B	107.9	Na1—O8—H8A	106 (3)
N1—C2—C1	114.4 (4)	Na1—O8—H8B	104 (3)
N1—C2—H2A	108.7	H8A—O8—H8B	120 (3)
			
O5—S1—O4—Na1	90 (2)	C5—N2—C7—N5	−178.9 (4)
O3—S1—O4—Na1	−38 (2)	C5—N2—C7—N4	0.8 (6)
C11—S1—O4—Na1	−154 (2)	C8—N5—C7—N2	4.3 (7)
O7—Na1—O4—S1	48 (2)	C8—N5—C7—N4	−175.4 (4)
O6^i^—Na1—O4—S1	−73 (2)	C6—N4—C7—N2	−1.9 (6)
O8—Na1—O4—S1	141.4 (19)	C6—N4—C7—N5	177.8 (4)
O6—Na1—O4—S1	−166 (2)	C7—N5—C8—C9	171.8 (4)
Cl1^ii^—Na1—O4—S1	119 (2)	C7—N5—C8—C13	−10.3 (7)
Na1^i^—Na1—O4—S1	−120 (2)	C13—C8—C9—C10	3.2 (7)
C5—N1—C2—C1	−76.6 (5)	N5—C8—C9—C10	−178.8 (4)
C3—N1—C2—C1	101.9 (4)	C8—C9—C10—C11	−2.2 (7)
O1—C1—C2—N1	−50.9 (5)	C9—C10—C11—C12	0.7 (6)
C5—N1—C3—C4	−94.4 (5)	C9—C10—C11—S1	176.5 (3)
C2—N1—C3—C4	87.0 (5)	O4—S1—C11—C10	−86.3 (4)
N1—C3—C4—O2	−61.9 (5)	O5—S1—C11—C10	33.7 (4)
C3—N1—C5—N2	−175.5 (4)	O3—S1—C11—C10	153.4 (3)
C2—N1—C5—N2	2.9 (6)	O4—S1—C11—C12	89.4 (4)
C3—N1—C5—N3	3.9 (6)	O5—S1—C11—C12	−150.5 (4)
C2—N1—C5—N3	−177.7 (3)	O3—S1—C11—C12	−30.8 (4)
C7—N2—C5—N1	179.5 (4)	C10—C11—C12—C13	−0.4 (7)
C7—N2—C5—N3	0.1 (7)	S1—C11—C12—C13	−176.2 (3)
C6—N3—C5—N1	−179.1 (4)	C9—C8—C13—C12	−2.9 (6)
C6—N3—C5—N2	0.3 (7)	N5—C8—C13—C12	179.3 (4)
C5—N3—C6—N4	−1.8 (7)	C11—C12—C13—C8	1.5 (7)
C5—N3—C6—Cl1	178.1 (3)	O7—Na1—O6—Na1^i^	171.9 (2)
C7—N4—C6—N3	2.6 (7)	O4—Na1—O6—Na1^i^	84.80 (11)
C7—N4—C6—Cl1	−177.4 (3)	O6^i^—Na1—O6—Na1^i^	0.0
Na1^ii^—Cl1—C6—N3	16.5 (4)	O8—Na1—O6—Na1^i^	−111.45 (12)
Na1^ii^—Cl1—C6—N4	−163.6 (2)	Cl1^ii^—Na1—O6—Na1^i^	156.93 (10)

Symmetry codes: (i) −*x*+2, −*y*+2, −*z*+1; (ii) −*x*+2, −*y*+2, −*z*.

### Hydrogen-bond geometry (Å, º)

**Table:**

*D*—H···*A*	*D*—H	H···*A*	*D*···*A*	*D*—H···*A*
O1—H1···N4^iii^	0.87 (2)	1.96 (3)	2.823 (5)	172 (5)
O2—H2···O3^iv^	0.86 (3)	1.94 (3)	2.772 (4)	165 (4)
O6—H6*A*···O5^v^	0.99	1.83	2.817 (4)	174
O6—H6*B*···N3^ii^	0.99	2.23	3.009 (5)	135
O7—H7*A*···O8^vi^	0.87 (2)	2.02 (3)	2.861 (5)	164 (4)
O7—H7*B*···O2^iv^	0.82 (2)	1.95 (2)	2.767 (4)	169 (5)
O8—H8*A*···O5^i^	0.80 (2)	2.03 (3)	2.797 (4)	162 (4)
O8—H8*B*···O3^v^	0.82 (2)	2.15 (3)	2.907 (5)	154 (4)
N5—H5···O1^ii^	0.90 (4)	2.01 (4)	2.828 (5)	151 (4)

Symmetry codes: (i) −*x*+2, −*y*+2, −*z*+1; (ii) −*x*+2, −*y*+2, −*z*; (iii) *x*, *y*−1, *z*; (iv) −*x*+1, −*y*+1, −*z*; (v) *x*+1, *y*, *z*; (vi) −*x*+2, −*y*+1, −*z*+1.
